# New Thinking on Neurodevelopment

**DOI:** 10.1289/ehp.114-a100

**Published:** 2006-02

**Authors:** Michael Szpir

The notion that some substances in the environment can damage the nervous system has an ancient history. The neurotoxicity of lead was recognized more than 2,000 years ago by the Greek physician Dioscerides, who wrote, “Lead makes the mind give way.” In the intervening millennia many other substances have been added to the list of known or suspected neurotoxicants. Despite this accumulation of knowledge, there is still much that isn’t understood about how neurotoxicants affect the developing brain, especially the effects of low-dose exposures. Today researchers are taking a hard look at low-dose exposures *in utero* and during childhood to unravel some of the mysteries of impaired neurodevelopment.

About 17% of school-age children in the United States suffer from a disability that affects their behavior, memory, or ability to learn, according to a study published in the March 1994 issue of *Pediatrics* by a team from the Centers for Disease Control and Prevention (CDC). The list of maladies includes attention deficit/hyperactivity disorder (ADHD), autistic spectrum disorders, epilepsy, Tourette syndrome, and less specific conditions such as mental retardation and cerebral palsy. All are believed to be the outcome of some abnormal process that unfolded as the brain was developing *in utero* or in the young child.

These disorders have an enormous impact on families and society. According to the 1996 book *Learning Disabilities: Lifelong Issues*, children with these disorders have higher rates of mental illness and suicide, and are more likely to engage in substance abuse and to commit crimes as adults. The overall economic cost of neurodevelopmental disorders in the United States is estimated to be $81.5–167 billion per year, according to a report published in the December 2001 issue of *EHP Supplements*.

Potentially even more disturbing is that a number of epidemiologic studies suggest that the incidence of certain disorders is on the rise. In the United States, the diagnosis of autistic spectrum disorders increased from 4–5 per 10,000 children in the 1980s to 30–60 per 10,000 children in the 1990s, according to a report in the August 2003 *Journal of Autism and Developmental Disorders*. Similarly, notes a report in the February 2002 issue of *CNS Drugs*, the diagnosis of ADHD grew 250% between 1990 and 1998. The number of children in special education programs classified with learning disabilities increased 191% between 1977 and 1994, according to an article in *Advances in Learning and Behavioral Disabilities, Volume 12*, published in 1998.

So what is going on? The short answer is that no one really knows. There’s not even consensus on what the soaring rates actually mean. Heightened public awareness could account for the surge in the numbers, or it may be that physicians are getting better at diagnosing the conditions. Some autism researchers believe the rise in that condition’s prevalence simply reflects changes in diagnostic criteria over the last 25 years. On the other hand, some scientists believe that the rates of neurodevelopmental disease are truly increasing, and that the growing burden of chemicals in the environment may play a role.

With that in mind, investigators are considering the effects of gene–environment interactions. A child with a mild genetic tendency toward a neurodevelopmental disorder might develop without clinically measurable abnormalities in the absence of environmental “hits.” However, children in industrialized nations develop and grow up in a veritable sea of xenobiotic chemicals, says Isaac Pessah, director of the University of California, Davis, Center for Children’s Environmental Health and Disease Prevention. “Fortunately,” he says, “most of us have a host of defense mechanisms that protect us from adverse outcomes. However, genetic polymorphisms, complex epistasis, and cytogenetic abnormalities could weaken these defenses and amplify chemical damage, initiating a freefall into a clinical syndrome.”

Pessah cites the example of autism. He says susceptibility for autism is likely conferred by several defective genes, no one of which can account for all the core symptoms of social disinterest, repetitive and overly focused behaviors, and problems in communication. Could multiple genetic liabilities and exposure to a chemically complex environment act in concert to increase the incidence and severity of the condition?

Despite the uncertainties, many scientists believe it would be wise to err on the side of caution when it comes to a research agenda. As Martha Herbert, a pediatric neurologist at Harvard Medical School, puts it, “Even though we may have neither consensus nor certainty about an autism epidemic, there are enough studies coming in with higher numbers that we should take it seriously. Environmental hypotheses ought to be central to research now. The physiological systems that have been harmed by environmental factors may also point to treatment targets, and this might be a great way to help the children.”

## The Parade of Neurotoxicants

Among the most intensely studied neurotoxicants are metals (lead, mercury, and manganese), pesticides, polychlorinated biphenyls (PCBs), and polybrominated diphenyl ethers (PBDEs). A number of these compounds were identified as neurotoxicants when individuals were exposed to high doses during occupational accidents or childhood poisonings. Scientists are now exploring the potential consequences of low-dose exposures, especially to children and fetuses. Epidemiologic studies play a central role, and these are often complemented by experimental work on animals and cell cultures. These days, researchers are looking not only at associations between toxicants and disease, but also at the underlying cellular and molecular mechanisms.

### Lead.

Studies dating to the 1970s show that children exposed to lead have deficits in IQ, attention, and language. In response, the CDC revised its limits for acceptable blood levels of the metal in several steps, from 60 micrograms per deciliter (μg/dL) in the 1960s to the current level of 10 μg/dL, set in 1991. But many scientists think that limit is still too high. A study reported in the September 2005 issue of *EHP* found that there were significant effects on a child’s IQ even when blood lead concentrations were below 10 μg/dL. Upon the July 2005 release of the *Third National Report on Human Exposure to Environmental Chemicals* by the CDC, Jim Pirkle, deputy director for science at the CDC’s Environmental Health Laboratory, stated, “There is no safe blood [lead] level in children.”

Several groups have also found evidence that lead exposure may shape a child’s social behavior. An article in the May 2000 issue of *Environmental Research* reports a strong correlation, dating back to 1900, between violent crime and the use of lead-based paint and leaded gasoline. The research complements studies by Herbert Needleman, a professor of psychiatry and pediatrics at the University of Pittsburgh School of Medicine, who found that bone lead levels in young males were correlated with aggression and criminality. “Lead is significantly associated with a risk for delinquency,” says Needleman. His research appeared in the November–December 2002 issue of *Neurotoxicology and Teratology* and the 7 February 1996 issue of *JAMA*.

Another new area of research links early lead exposure to changes in the aging brain. Nasser Zawia, an associate professor of pharmacology and toxicology at the University of Rhode Island, Kingston, and his colleagues found increased expression of amyloid precursor protein (APP) and its product, β -amyloid (which is a hallmark of Alzheimer disease), in aging rats that were exposed to lead shortly after birth. In contrast, old rats that were exposed to lead did not show an increased expression of APP and β -amyloid. The work, published in the 26 January 2005 issue of *The Journal of Neuroscience*, suggests that early exposure to lead can “reprogram” gene expression and regulation later in life. According to Zawia, preliminary research also shows that “monkeys exposed to lead as infants exhibit similar molecular changes as well as exaggerated Alzheimer’s pathology.”

### Mercury.

The current Environmental Protection Agency (EPA) reference dose for methylmercury (an organic, toxic form of mercury) is 0.1 micrograms per kilogram per day (μg/kg/day). Humans are exposed to methylmercury primarily through consumption of contaminated fish; a good 70% of this contamination comes from anthropogenic sources such as emissions from coal-fired power plants. High-level exposure to methylmercury in the womb is linked to a number of impairments, including mental retardation, cerebral palsy, seizures, deafness, blindness, and speech difficulties. An article in the May 2005 issue of *EHP* puts the economic cost to the United States of methylmercury-induced toxicity (in terms of lost productivity) at $8.7 billion annually.

The effects of low-dose exposures are not so apparent. Two large epidemiologic studies of fishing populations in the Faroe Islands and the Seychelles have produced conflicting results regarding low-dose effects. Both studies sought to examine the association between methylmercury exposure and neurodevelopment in children whose mothers ate contaminated seafood during pregnancy.

The leader of the Faroe Islands study, Philippe Grandjean, an adjunct professor of environmental health at the Harvard School of Public Health, and his colleagues reported in the November 1997 issue of *Neurotoxicology and Teratology* that 7-year-old Faroese children had significant cognitive deficits and neurological changes after prenatal exposure to methylmercury. Grandjean’s team followed up on the children at age 14. According to a report in the February 2004 issue of *The Journal of Pediatrics,* the children continued to have problems, including neurological changes and decreased nervous control of the heart.

In contrast, the authors of the Seychelles study found little evidence of lasting harm on a cohort of 66-month-old children, according to their report in the 26 August 1998 issue of *JAMA*. A follow-up study, published in the 17 May 2003 issue of *The Lancet,* similarly found no lasting effects on language, memory, motor skills, or behavioral function when the children were 9 years old.

The different outcomes of the two studies are puzzling because the children of both populations appeared to be exposed to similar amounts of methylmercury. Several explanations have been proposed, including the possibility that genetic differences between the populations may alter their relative predispositions to harm from mercury exposure. The source of methylmercury is also different in the two populations. The Faroese are exposed primarily through the consumption of pilot whale meat, whereas the Seychelles population relies heavily on ocean fish. According to Gary Myers, a professor of neurology and pediatrics at the University of Rochester Medical Center and one of the principal investigators of the Seychelles study, whale meat contains many other contaminants (including PCBs) besides methylmercury. “There is also evidence,” he says, “that the effects of concomitant PCB and mercury exposure are synergistic.”

Researchers continue to look at whether there is a danger from methylmercury at the levels of exposure achieved by fish consumption. Another layer of uncertainty was added with findings published in the October 2005 issue of *EHP* showing that fish consumption during pregnancy appeared to boost infant cognition—but only as long as mercury intake, as measured in maternal hair, wasn’t too high.

The question of whether low levels of mercury are harmful has also manifested itself in a controversy over the use of vaccines containing thimerosal, a preservative. Although thimerosal was removed from many of these vaccines in 2001, children that were immunized before that date could have received a cumulative dose of more than 200 μg/kg of mercury with the routine complement of childhood vaccinations, according to a study in the May 2001 issue of *Pediatrics*. Thimerosal is nearly half ethyl-mercury by weight. Because ethylmercury is an organic form of mercury, there is some suspicion that it acts like methylmercury in the brain, although research published in the August 2005 issue of *EHP* suggests that the two forms differ greatly in how they are distributed through and eliminated from the brain. Developing countries continue to use pediatric vaccines that contain thimerosal. In the United States, thimerosal is still present in influenza vaccines, which the CDC recommends be given to pregnant women and children aged 6–23 months.

Advocacy groups, such as SafeMinds, have suggested that the decades-long rise in the diagnosis of autism is related to the presence of thimerosal in vaccines. In May 2004, however, the Institute of Medicine (IOM) issued a report, *Immunization Safety Review: Vaccines and Autism*, stating that several epidemiological studies published since 2001 “consistently provided evidence of no association” between thimerosal-containing vaccines and autism. However, the IOM’s report has been severely criticized by a number of advocacy groups, including the National Autism Association, for relying too heavily on a specific set of epidemiologic data while dismissing clinical evidence and other epidemiologic studies that showed evidence of a link.

Despite the assurances of the IOM, some scientists continue to explore the mechanisms underlying the potential neurotoxic effects of thimerosal. In the January 2005 issue of *NeuroToxicology*, S. Jill James, a professor of pediatrics at the University of Arkansas for Medical Sciences, and her colleagues report that the neuronal and glial cell toxicity of methylmercury and ethylmercury (as dosed via thimerosal) are both mediated by the depletion of the antioxidant peptide glutathione. Of the two cell types, neurons were found to be particularly susceptible to ethylmercury-induced glutathione depletion and cell death, according to James, and pretreatment of the cells with glutathione reduced these effects. Other studies by James and her colleagues, reported in the December 2004 issue of the *American Journal of Clinical Nutrition*, showed that autistic children had lower levels of glutathione compared to normal controls, and may therefore have had a significant reduction in the ability to detoxify reactive oxygen species.

James says the abnormal profile “suggests that these children may have an increased vulnerability to pro-oxidant environmental exposures and a lower threshold for oxidative neurotoxicity and immunotoxicity.” Speaking at the XXII International Neurotoxicology Conference in September 2005, she presented evidence that multiple genetic polymorphisms affecting glutathione pathways may interact to produce a chronic metabolic imbalance that could contribute to the development and clinical symptoms of autism. Her paper in the *American Journal of Clinical Nutrition* reported that low glutathione levels in many autistic children were reversible with targeted nutritional intervention, but the ramifications of this finding are still unclear.

### Manganese.

As an essential nutrient, manganese is required for normal development; the reference dose for manganese is 0.14 mg/kg/day. Chronic occupational exposure to high levels of this metal is associated with manganism, a condition reminiscent of Parkinson disease that is characterized by tremors, rigidity, and psychosis. The illness is seem primarily among miners.

Animal studies published in the August 2005 issue of *Neurotoxicology* by David Dorman, director of the division of biological sciences at the CIIT Centers for Health Research in Research Triangle Park, North Carolina, suggest that the fetus is protected to a certain extent from maternally inhaled manganese. According to Dorman, children are exposed to manganese primarily by ingesting it, but he knows of no link between childhood exposure to manganese and later Parkinson disease.

Nevertheless, because manganese affects the adult brain, people suspect that the developing brain may be even more susceptible to harm from this metal, and recent research has unveiled a new cause for concern: In the January 2006 issue of *EHP*, child psychiatry professor Gail Wasserman and colleagues from Columbia University reported that Bangladeshi children who drank well water with high concentrations of naturally occurring manganese had diminished intellectual function. The researchers noted that the bioavailability of manganese in water is higher than that of manganese in food. They also pointed out that about 6% of U.S. wells have a high enough manganese content to potentially put some children at risk for diminished intellectual function.

The cellular and molecular mechanisms of manganese neurotoxicity are not well understood. The dopaminergic system in the basal ganglia, which is affected in Parkinson disease, may be involved, but this hypothesis is controversial. Tomás Guilarte, a professor of molecular neurotoxicology at the Johns Hopkins Bloomberg School of Public Health, described research on these systems in nonhuman primates at the XXII International Neurotoxicology Conference. According to Guilarte, unpublished positron-emission tomography studies of the basal ganglia show that “manganese does appear to have an effect on dopaminergic neurons.” Guilarte found that the more manganese the animals received, the less dopamine was released through the actions of amphetamine (which is used to induce the release of the neurotransmitter). “This does not mean that manganese causes Parkinson’s disease, merely that it has an effect on those neurons,” he says. This is the first report of an *in vivo* effect on dopamine release by manganese.

### PCBs, PBDEs, and pesticides.

Many chemicals raise concerns because of their persistence in the environment and their tendency to bioaccumulate in animal tissues. They are typically synthetic molecules that were designed for use in everyday products, such as electrical equipment, computers, furniture, and pesticides.

PCBs appear to be present in all parts of the food chain, and humans are exposed to these molecules primarily through the ingestion of animal fat. The toxicity of these chemicals was first recognized after mass poisonings in Japan in 1968 and Taiwan in 1979. Children born to women who had ingested contaminated cooking oil in Taiwan had a number of developmental abnormalities, including psychomotor delay and lower scores on cognitive tests, according to a report in the 15 July 1988 issue of *Science*.

Since those earlier observations, several studies have described a connection between prenatal exposure to PCBs and delayed cognitive development and lower IQ. For example, a study in the 10 November 2001 *Lancet* reports those infants and young children exposed to PCBs through breast milk scored lower on tests of psychomotor and mental development. The mothers were exposed to normal background levels of PCBs in Europe. In response to such studies, the U.S. Food and Drug Administration set tolerance levels for PCBs in a number of consumer products, such as milk and manufactured dairy products (1.5 parts per million), poultry (3.0 parts per million), and baby food (0.2 part per million).

PBDEs are widely used as flame retardants in consumer products. The effects of PBDEs on humans is not clear, but animal toxicity studies described in volume 183 (2004) of *Reviews of Environmental Contaminants and Toxicology* show that PBDEs can cause permanent learning and memory impairments, hearing deficits, and behavioral changes. There is a growing concern about PBDEs because they appear to be accumulating in human tissues. Andreas Sjödin, a toxicologist at the CDC, and colleagues found a trend toward increasing concentrations of PBDEs in human serum taken from sample populations in the southeastern United States from 1985 through 2002, and in Seattle, Washington, from 1999 through 2002. This report appears in the May 2004 *EHP*. Several studies have also discovered PBDEs in human breast milk. The current EPA reference dose for PBDEs is 2 mg/kg/day.

As for pesticides, it’s been suggested by zoologist Theo Colborn of the University of Florida that every child conceived today in the Northern Hemisphere is exposed to these chemicals from conception through gestation and beyond. Some pesticides appear to be more harmful than others, and so the reference dose varies somewhat from one compound to another.

The effects of pesticides on the developing brain have been investigated in human epidemiologic studies and in laboratory experiments with animals. Vincent Garry, a professor of environmental medicine at the University of Minnesota, and his colleagues found that children born to applicators of the fumigant phosphine were more likely to display adverse neurological and neurobehavioral developmental effects. The herbicide glyphosate was also linked to neurobehavioral effects, according to the same report, which appeared in the June 2002 issue of *EHP Supplements*. Another epidemiologic study, reported in the March 2005 issue of *NeuroToxicology,* showed that women who were exposed to organophosphate pesticides in an agricultural community in California had children who displayed adverse neurodevelopmental effects, and that higher levels of pesticide metabolites in maternal urine were associated with abnormal reflexes in the women’s newborn children.

Many PCBs, PBDEs, and pesticides are the subject of the 2001 Stockholm Convention on Persistent Organic Pollutants, which became international law in May 2004. The goal of the treaty is to “rid the world of PCBs, dioxins and furans, and nine highly dangerous pesticides,” according to the United Nations Environment Programme. Implementation of the treaty has significant practical challenges, however, including the difficulty of eliminating one persistent pollutant without creating another (for example, when burning PCBs yields by-products such as dioxins and furans).

## Not Immune to Harm

Exposure to a neurotoxicant may not be the only way to disrupt the natural growth of the brain. Scientists are now looking at the subtle physiological effects of immunotoxicants and infectious agents on biological events during development.

It turns out that mothers who experience an infection during pregnancy are at a greater risk of having a child with a neurodevelopmental disorder such as autism or schizophrenia. For example, prenatal exposure to the rubella virus is associated with neuromotor and behavioral abnormalities in childhood and an increased risk of schizophrenia spectrum disorders in adulthood, according to an article in the March 2001 issue of *Biological Psychiatry*. Rubella has also been linked to autism: some 8–13% of children born during the 1964 rubella pandemic developed the disorder, according to a report in the March 1967 *Journal of Pediatrics*. The same study also noted a connection between the rubella virus and mental retardation.

Some epidemiologic studies have found an increased risk of schizophrenia among the children of women who were exposed to the influenza virus during the second trimester of pregnancy, according to a report in the February 2002 *Current Opinion in Neurobiology*. In the August 2004 *Archives of General Psychiatry,* Ezra Susser, head of epidemiology at Columbia University’s Mailman School of Public Health, and his colleagues reported that the risk of the mental disorder was increased sevenfold if the schizophrenic patient’s mother had influenza during her first trimester of pregnancy. A prospective birth cohort study in the April 2001 *Schizophrenia Bulletin* found that second trimester exposure to the diphtheria bacterium also significantly increased the risk of schizophrenia.

How might infectious agents cause these disorders? According to John Gilmore, a professor of psychiatry at the University of North Carolina at Chapel Hill, maternal infections during pregnancy can alter the development of fetal neurons in the cerebral cortex of rats. The mechanism is far from clear, but signaling molecules in the mother’s immune system, called cytokines, have been implicated. Speaking at the XXII International Neurotoxicology Conference, Gilmore described *in vitro* experiments showing that elevated levels of certain cytokines—interleukin-1β , interleukin-6 and tumor necrosis factor–alpha (TNF-α)—reduce the survival of cortical neurons and decrease the complexity of neuronal dendrites in the cerebral cortex. “I believe that the weight of the data to date indicates [that the maternal immune response] can have harmful effects,” says Gilmore.

Inflammatory responses in the mother may not be the only route to modifying the fetal brain. The University of California, Davis, Center for Children’s Environmental Health and Disease Prevention is conducting a large study of autistic children in California called CHARGE (Childhood Autism Risks from Genetics and the Environment), which suggests that the child’s immune system may also be involved. According to Pessah, the study principal investigator, children with autism appear to have a unique immune system. “Autistic children have a significant reduction in plasma immunoglobulins and a skewed profile of plasma cytokines compared to other children,” he says. “We think that an immune system dysfunction may be one of the etiological cores of autism.”

He continues, “We know that many of the things that kids are exposed to these days are immunotoxicants. . . . We have evidence that ethylmercury and thimerosal alter the signaling properties of antigen-presenting cells, known as dendritic cells, at nanomolar levels.” Since each dendritic cell can activate 250 T cells, any dysregulation will be magnified, he says. “Add to that a genetic abnormality in processing immune information, and there could be a problem.”

Such problems might extend to the central nervous system. The brains of individuals who have a neurodevelopmental disorder also show evidence of inflammation. In the January 2005 issue of the *Annals of Neurology*, Carlos Pardo, an assistant professor of neurology and pathology at the Johns Hopkins University School of Medicine, and his colleagues report finding high levels of inflammatory cytokines (interleukin-6, interleukin-8, and interferon-γ) in the cerebrospinal fluid of autistic patients. Glial cells, which serve as the brain’s innate immune system, are the primary sources of cytokines in the central nervous system. So it may not be surprising that Pardo’s team also discovered that glia are activated—showing both morphological and physiological changes—in postmortem brains of autistic patients.

The recognition that the immune system is involved in neurodevelopmental disorders is changing people’s perceptions of these conditions. “Historically, scientists have focused on the role of neurons in all kinds of neurological diseases,” Pardo says, “but they have generally been ignoring the [glia].” He adds, “In autism, it could be that the [glia] are responding to some external insult, such as an infection, an intrauterine injury, or a neuro-toxicant.”

According to Pardo, it’s still not clear whether the neuroimmune responses associated with autism contribute to the dysfunction of the brain or whether they are secondary reactions to some neural abnormality. “John Gilmore’s work [showing that cytokines can be harmful to brain cells] is quite interesting and important,” he says. “However, *in vitro* studies may produce results that don’t reflect what occurs under *in vivo* conditions. Cytokines like TNF-αmay be beneficial for some neurobiological functions at low concentrations, but may be extremely neurotoxic at high concentrations.”

## Lending Brain Power to Exposure Assessment

The medical and scientific communities recognize the colossal challenges involved in identifying the ultimate causes of neurodevelopmental disorders. This is complicated by the sheer numbers of potential exposures involved. More than 67% of the nearly 3,000 chemical compounds produced or imported in amounts exceeding 1 million pounds per year have not been examined with even basic tests for neurotoxicity, according to *Toxic Ignorance*, a 1997 analysis by Environmental Defense.

In the past few years, several large projects have been proposed, and funding by the NIH has been increased. For example, the NIH boosted its support for autism research from $22 million in 1997 to $100 million in 2004. In 2001, the NIEHS and the EPA jointly announced the creation of four new children’s environmental health research centers (including the one at the University of California, Davis), which focus primarily on neurodevelopmental disorders. More recently, the proposed multibillion-dollar National Children’s Study, which is cosponsored by the Department of Health and Human Services and the EPA, has been designed to follow nearly 100,000 children over the course of 21 years. The investigators plan to study the effects of environmental factors on children’s growth and development, including impacts on learning, behavior, and mental health. Study investigators hope to enroll the first participants in early 2007.

Scientists also see the need for designing better studies. In neurodevelopmental studies, as in any other field, the quality of a study is only as good as all of its parts. Jean Harry, head of the NIEHS Neurotoxicology Group, says, “You can have a valid assessment of behavior, but in the absence of good exposure data, a causative association with environmental factors will be compromised.”

In a bid to address the difficulties faced by epidemiologic studies that look for neurodevelopmental effects from *in utero* chemical exposure, a working group of 20 experts gathered in September 2005 under the auspices of the Penn State Hershey Medical Center, coincident with the XXII International Neurotoxicology Conference. The goal of their day-long session was to develop a scheme of best practices for the design, conduct, and interpretation of future investigations, as well as the practical inclusion of new technologies, such as imaging.

At one point in the dialogue, the group recognized that perhaps the greatest challenge in these studies was determining how to evaluate *in utero* exposures to environmental chemicals. “Quite often the very nature of epidemiological studies limits the ability to perform accurate exposure assessments,” says Harry, who was part of the expert group. “Such exposures may have occurred in the distant past, they may have been unknown, or they may have been in conjunction with many other compounds.”

The group therefore recommended that actual measurements, even if indirect, are better than methods based on subject recall. It also recommended that a well-defined hypothesis should form the foundation of *in utero* studies for assessing neurodevelopmental outcomes. “[These and other] conclusions will move the science forward by describing methods that should improve interstudy comparisons, and they offer ways in which research results should be reported to the scientific and medical communities,” says Judy LaKind, an adjunct associate professor of pediatrics at the Hershey Medical Center and a member of the workshop steering committee. The complete workshop report will be published in an upcoming issue of *NeuroToxicology*.

## Imagining the Big Picture

The challenges of addressing neurodevelopmental disorders are more than scientific. The difficulties come together at a crossroads where the communication of knowledge, the treatment of patients, and the regulation of potentially toxic chemicals meet. Says Herbert, “Evidence-based medicine has not yet developed standards for assessing, or practices for treating, the impacts of chronic, multiple low-dose exposures.” Rather than waiting, she says, patients and parents of patients are turning to alternative medicine to address their concerns.

That’s not always a good thing, especially when patients and parents may be misinformed. Kathy Lawson, director of the Healthy Children Project at the Learning Disabilities Association of America, says there is a disconnect between scientific knowledge and the public’s awareness of ways to reduce the incidence of some disorders. “In my visits to various organizations, I’ve discovered that people are completely unaware that there is a connection between environmental toxicants and their health,” she says. “Even pediatricians often don’t know about these things,” she adds.

Educating the public is only part of the solution. Elise Miller, executive director of the nonprofit Institute for Children’s Environmental Health, thinks that federal regulatory agencies do not adequately protect children’s health. “The Toxic Substances Control Act, which was passed thirty years ago, needs a major overhaul to ensure neurotoxicants and other chemicals are prioritized, screened, and tested properly,” she says. “Currently, there are too many chemicals on the market and in the products we use every day for which there is no toxicity data.”

Some politicians agree with these sentiments. In July 2005, Senator Frank R. Lautenberg (D–NJ) introduced the Child, Worker, and Consumer Safe Chemicals Act, which initially calls for chemical manufacturers to provide health and safety information on the chemicals used in certain consumer products, among them baby bottles, water bottles, and food packaging. If passed into law, the bill, coauthored by Senator James Jeffords (I–VT), would require all commercially distributed chemicals to meet the new safety measures by 2020.

The human brain is often touted as the most complex structure in the known universe. The developmental process that produces this remarkable entity may also be among the most delicate in nature. As one scientist put it, “The brain doesn’t like to be jerked around.” That kind of fragility makes it difficult for scientists to untangle genetic influences from what often may be subtle environmental assaults. Even so, the catalogue of harmful environmental agents will undoubtedly continue to grow as scientists learn more about the interactions between the developing brain and its environment. The hope is that enough good minds will use that catalogue to create a future with healthier brains and more peace of mind for parents and society alike.

## Figures and Tables

**Figure:**
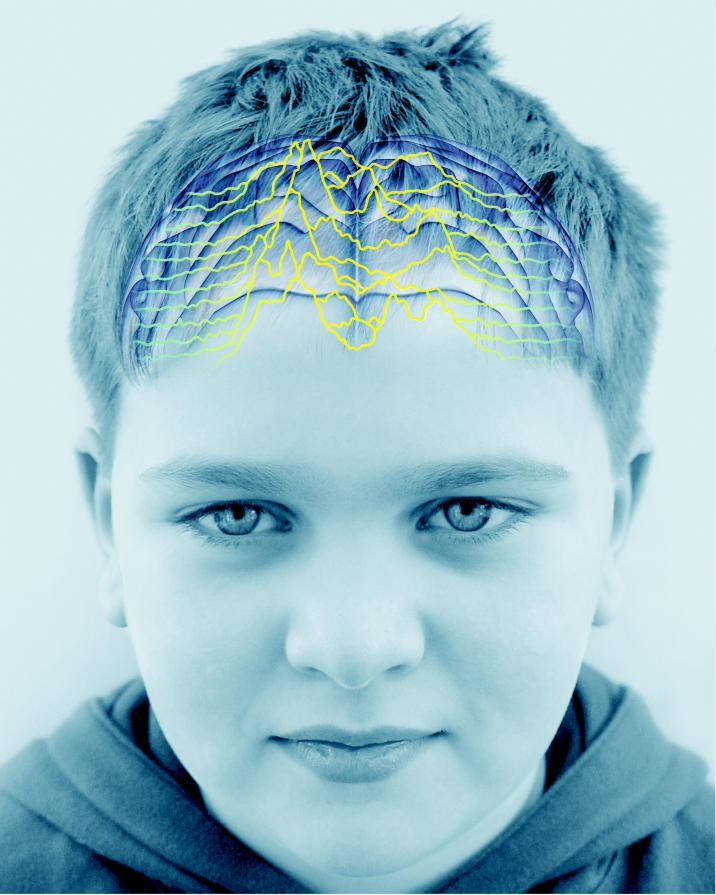


**Figure:**
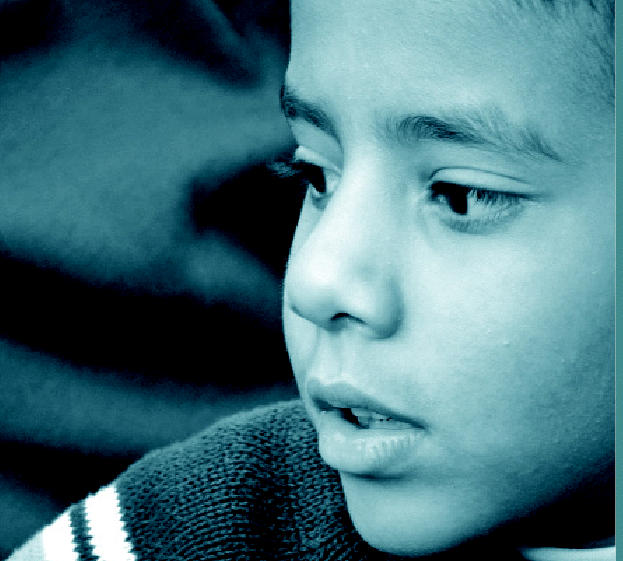
17 Percentage of school-age children in the United States who suffer from a disability that affects their behavior, memory, or ability to learn. Pediatrics, March 1994

**Figure:**
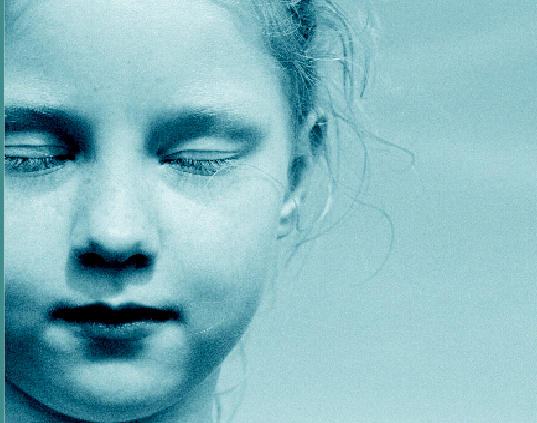
81.5 to 167 Annual cost in billions of dollars for neurodevelopmental disorders in the United States. EHP Supplements, December 2001

**Figure:**
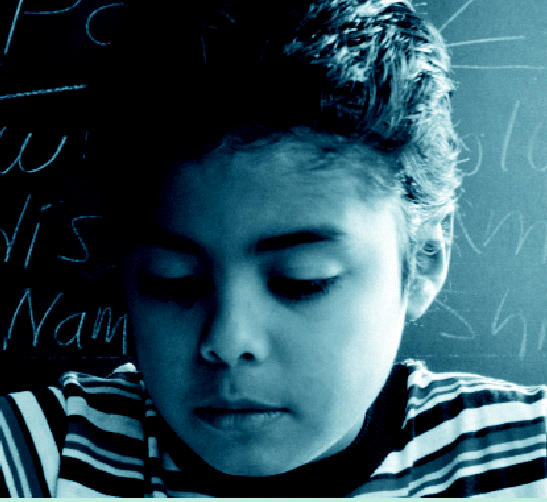
4–5 to 30–60 Increase from the 1980s to the 1990s in the number of U.S. children per 10,000 diagnosed with autistic spectrum disorders. Journal of Autism and Developmental Disorders, August 2003

**Figure:**
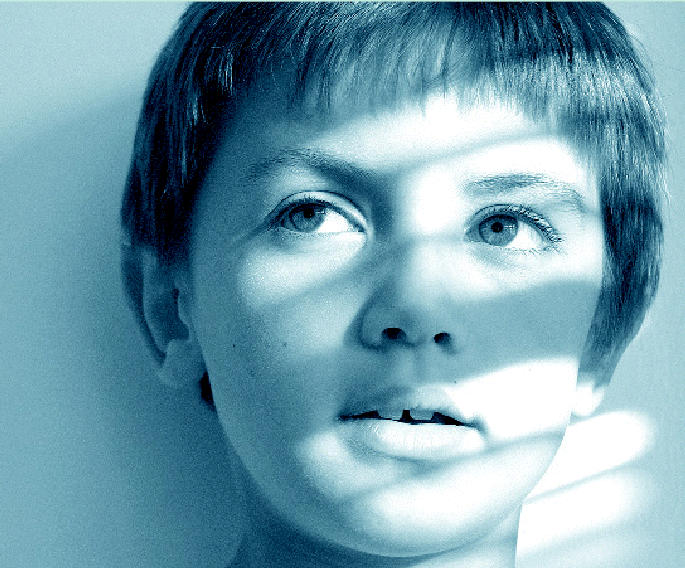
250 Percentage of increase in the number of U.S. children diagnosed with ADHD between 1990 and 1998. CNS Drugs, February 2002

**Figure:**
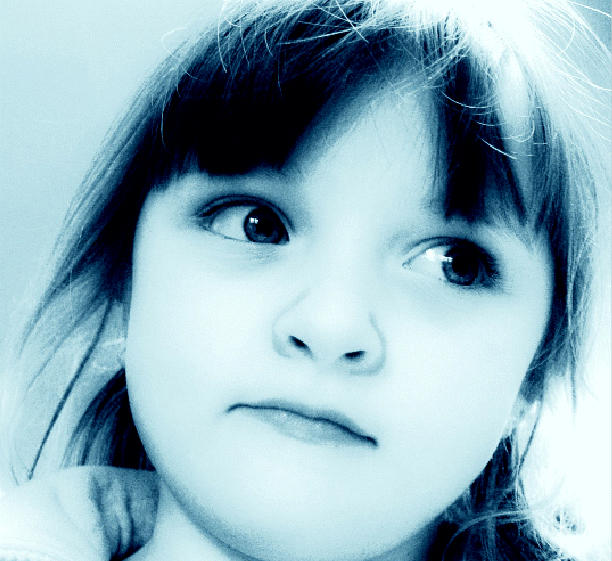
191 Percentage of increase in the number of U.S. children in special education programs classified with learning disabilities between 1977 and 1994. Advances in Learning and Behavioral Disabilities, Volume 12, 1998

**Figure:**
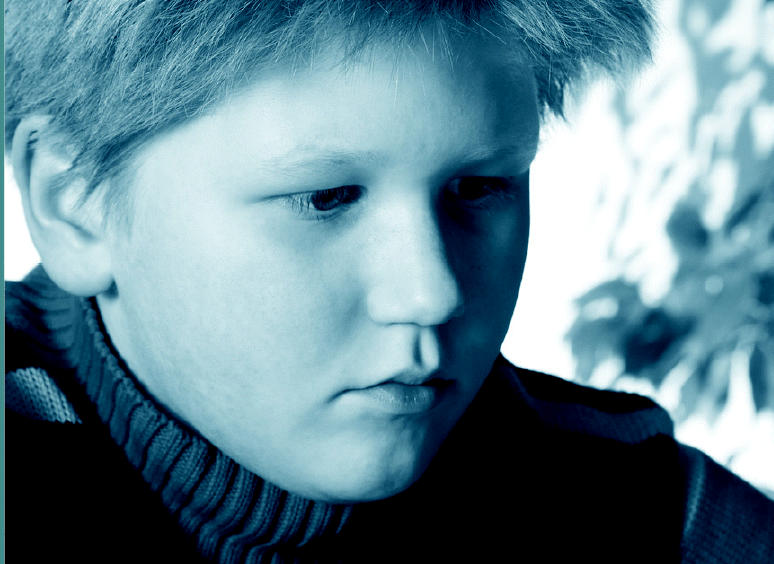
83 Percentage of decrease in the CDC acceptable level for lead in blood from 1960 to the current level, set in 1991. EHP, September 2005

**Figure:**
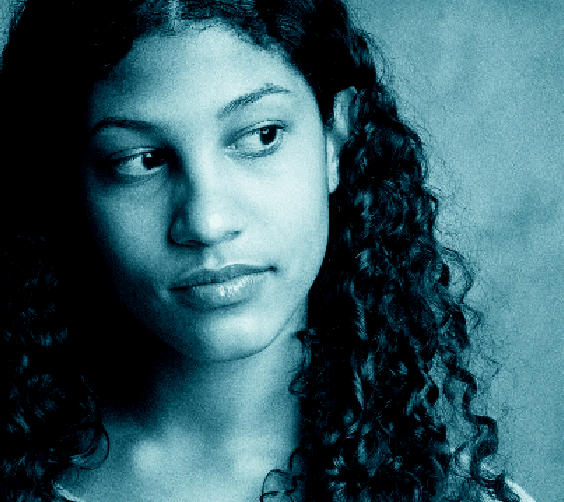
8.7 Annual cost in billions of dollars of methylmercury-induced toxicity (in terms of lost productivity). EHP, May 2005

**Figure:**
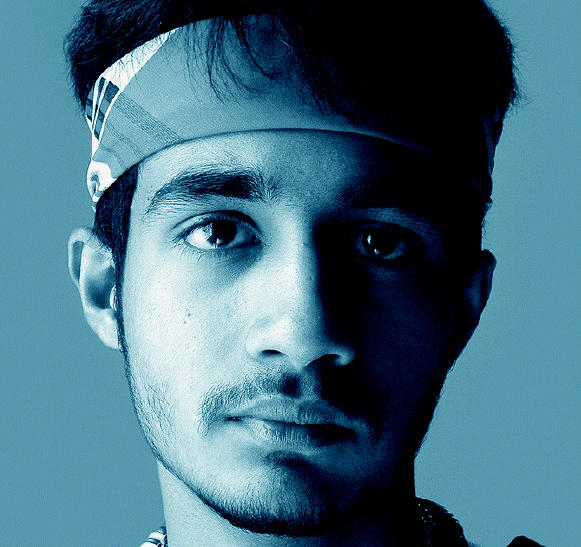
6 Percentage of U.S. wells with a high enough manganese content to potentially put some children at risk for diminished intellectual function. EHP, January 2006

**Figure:**
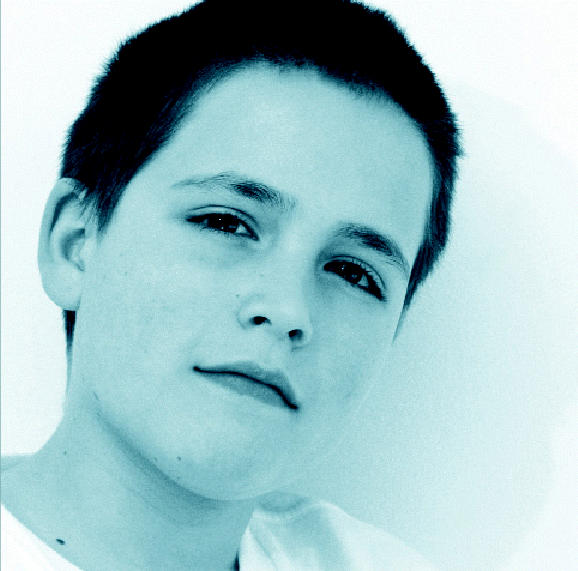
7-fold The increased risk of schizophrenia in offspring if the mother had influenza during her first trimester of pregnancy. Archives of General Psychiatry, August 2004

**Figure:**
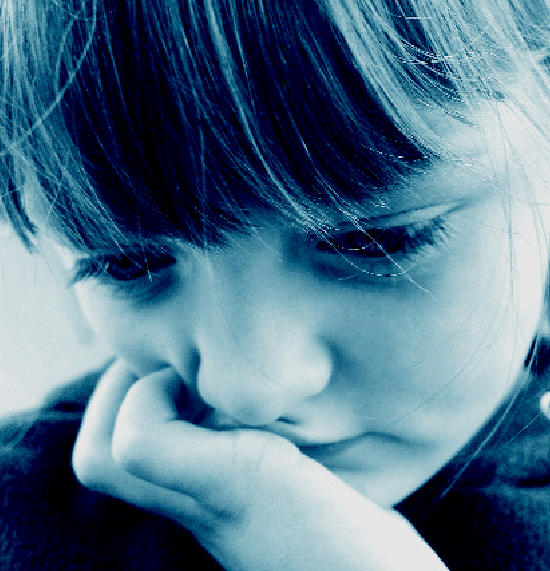
67 Percentage of high-production-volume chemicals produced in or imported into the United States that have not been examined for neurotoxicity. Toxic Ignorance, 1997

